# Gene therapy in PIDs, hemoglobin, ocular, neurodegenerative, and hemophilia B disorders

**DOI:** 10.1515/biol-2021-0033

**Published:** 2021-05-03

**Authors:** Arome Solomon Odiba, Nkwachukwu Oziamara Okoro, Olanrewaju Ayodeji Durojaye, Yanjun Wu

**Affiliations:** Molecular Biology Laboratory, National Engineering Research Center for Non-food Biorefinery, Guangxi Academy of Sciences, Nanning, China; Department of Biochemistry, College of Life Science and Technology, Guangxi University, Nanning, China; Department of Molecular Genetics and Biotechnology, University of Nigeria, Nsukka, Nigeria; Department of Biochemistry, University of Nigeria, Nsukka, Nigeria; Department of Pharmaceutical and medicinal Chemistry, University of Nigeria, Nsukka, Nigeria; Department of Biochemistry and Molecular Biology, University of Science and Technology of China, Hefei, Anhui, China; Animal Genetics, Breeding and Reproduction, College of Animal Science and Technology, Guangxi University, Nanning, 530004, China; Institute for Laboratory Animal, Guizhou University of Traditional Chinese Medicine, Guiyang, 550025, China; Department of Pharmaceutical and Medicinal Chemistry, College of Life Science and Technology, Guangxi University, Nanning, China

**Keywords:** clinical trials, gene therapy, hemoglobin, hemophilia B, neurodegenerative, ocular

## Abstract

A new approach is adopted to treat primary immunodeficiency disorders, such as the severe combined immunodeficiency (SCID; e.g., adenosine deaminase SCID [ADA-SCID] and IL-2 receptor X-linked severe combined immunodeficiency [SCID-X1]). The success, along with the feasibility of gene therapy, is undeniable when considering the benefits recorded for patients with different classes of diseases or disorders needing treatment, including SCID-X1 and ADA-SCID, within the last two decades. β-Thalassemia and sickle cell anemia are two prominent monogenic blood hemoglobin disorders for which a solution has been sought using gene therapy. For instance, transduced autologous CD34+ HSCs via a self-inactivating (SIN)-Lentivirus (LV) coding for a functional copy of the β-globin gene has become a feasible procedure. adeno-associated virus (AAV) vectors have found application in ocular gene transfer in retinal disease gene therapy (e.g., Leber’s congenital amaurosis type 2), where no prior treatment existed. In neurodegenerative disorders, successes are now reported for cases involving metachromatic leukodystrophy causing severe cognitive and motor damage. Gene therapy for hemophilia also remains a viable option because of the amount of cell types that are capable of synthesizing biologically active FVIII and FIX following gene transfer using AAV vectors *in vivo* to correct hemophilia B (FIX deficiency), and it is considered an ideal target, as proven in preclinical studies. Recently, the clustered regularly interspaced palindromic repeats (CRISPR)/CRISPR-associated protein 9 gene-editing tool has taken a center stage in gene therapy research and is reported to be efficient and highly precise. The application of gene therapy to these areas has pushed forward the therapeutic clinical application.

## 1 Background

**Figure 1:**
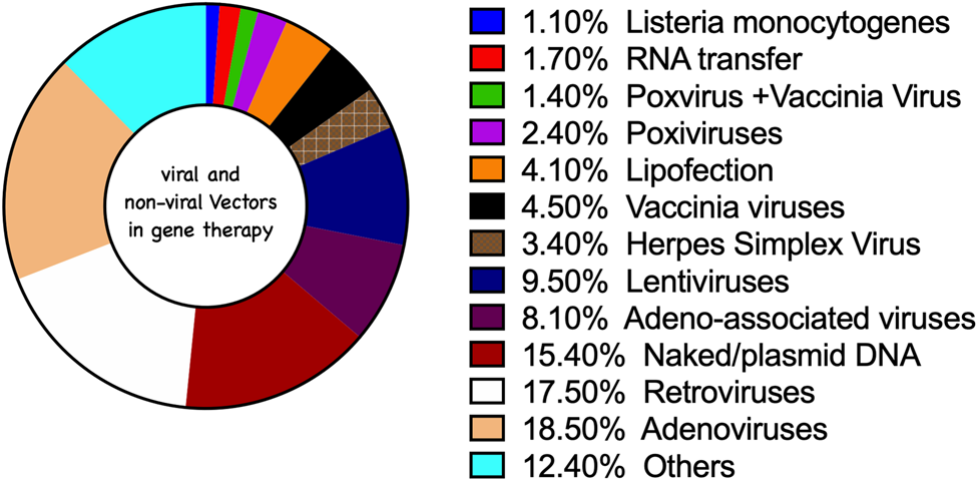
Viral and nonviral vectors used in gene therapy. Data sourced from: www.wiley.co.uk/genmed/clinical, 2018.

Molecular biology and biotechnology tools remained important elements in gene therapy. Gene editing/modification (replacement, insertion, and deletion) largely characterizes this field of biological sciences. The idea of gene therapy was implemented clinically about three decades ago as an alternative to the limitations of pharmacotherapy. Approximately 3,000 known clinical trials are on record (Table 1). Some limitations are associated with gene therapy; and hence, the need to improve on the current strategies. This has resulted in sophisticated tools using viral and nonviral vectors (Figure 1). Although most of the gene therapy studies are directed toward cancer worldwide (http://www.wiley.com/legacy/wileychi/genmed/clinical/), other areas of notable disease require the gene therapy approach; and these include primary immunodeficiency disorders (PIDs), hemoglobin, hemophilia B, ocular, and neurodegenerative disorders. Gene therapy encompasses the introduction of new therapeutic genes, as DNA segments, modification of existing genes, or the introduction of RNA into cells, with the aim of preventing, treating, or curing disorders and diseases in order to restore or add gene expression. Diseases such as diabetes, Parkinson’s disease, heart failure, cancers, and neurodegenerative and metabolic disorders have been well managed through gene therapy [1].

**Table 1:** Indications (disease conditions) addressed by gene therapy clinical trials in order of descending percentage hierarchy

Indications	Gene therapy clinical trials	
	Number	Percentage
Cancer diseases	1,590	64.6
Monogenic diseases	259	10.5
Infectious diseases	182	7.4
Cardiovascular diseases	178	7.2
Healthy volunteers	54	2.2
Neurodegenerative diseases	45	1.8
Others	56	2.3
Gene marking	50	2
Ocular diseases	34	1.4
Inflammatory diseases	15	0.6
Total	2,463	

Data sourced from: www.wiley.co.uk/genmed/clinical on 2nd June, 2017.

These strides are, however, not devoid of challenges that include recognizing the protein on the viral capsid by host immune system; delineating it as an antigen, though this has been managed through the development of elaborate technologies; and shielding the protein sites from eliciting inflammatory activities. Hence, this produces excellent safety profiles even for *in vivo* gene therapy [2]. A key advantage of this protocol is that adverse reactions and side effects are reduced to a feasible minimum, and it also provides room for the administration of the desired concentration. Therapeutic gene treatment also holds healthier potentials for use in managing diseases such as hemoglobinopathies, cancer immunotherapies, hemophilia B, ocular diseases, neurodegenerative diseases [3], immunological and metabolic disorders, and hematological diseases that have eluded cure, in resistance to the conventional treatment options [4,5]. Rapid improvements are recorded every year. Many phases of clinical and experimental trials are in the pipeline to improve on all the factors surrounding the anticipated accomplishments in gene therapy. It is, however, very pertinent to keep the medical and research community abreast of the most recent developments and redirections discussed under some of the systematic headings in the selected areas of PIDs, hemoglobin, ocular, neurodegenerative, and hemophilia B disorders as addressed in this study. We further briefly highlight the application of the more recent gene-editing tool, clustered regularly interspaced palindromic repeats (CRISPR)/CRISPR-associated protein 9 (CRISPR-cas9), but will be discussed in greater detail in the future reviews.

## 2 The path to treating PIDs

Records have been documented for many monogenic disorders (inheritable disorders that are a result of a single-defective gene on the autosomes) that require treatment using gene therapy. These are caused by a mutation in a single gene. Examples include cystic fibrosis, sickle cell disease, Wiskott–Aldrich syndrome (WAS), chronic granulomatous disease (CGD), Tay–Sachs disease, polycystic kidney disease, and PIDs, which comprise a class of rare, inheritable disorders of the immune system. A typical instance includes the severe combined immunodeficiency ([SCID] e.g., adenosine deaminase SCID [ADA-SCID] and IL-2 receptor X-linked severe combined immunodeficiency [SCID-X1]) [6]. The single most common strategy in dealing with these forms of diseases has been the genetic modification of hematopoietic stem cell (HSC), which has proven efficacious over the years. For example, HSC can be transplanted from a donor that has a similar and compatible HLA to a beneficiary (patient) to treat PID. Over 90% success has been recorded in such trials [7]. Substantial feasibility has been documented for the *ex vivo* delivery of a single gene into the isolated HSC, followed by transplant, with very well-established protocols. However, because of the complications associated with mismatch and incompatibility with some donors and their recipients, it is considered more expedient to modify the patient’s own HSC and readminister to the patient. Therefore, the donor is the recipient, and this factor rules out compatibility complications.

The phenotypic manifestation of the CGD is that the obviously mature phagocytes cannot carry out their function of killing the ingested microorganisms, potentially leading to serious inflammation. p22phox, gp91phox, p67phox, p47phox, and p40phox are all genes coding for the NADPH oxidase complex of the phagocytes, and it is in this set of genes that mutations occur, thereby impairing their function [8]. The catalytic subunit gp91phox coded by the cytochrome b (558) gene (CYBB) is located on the X-chromosome (X-CGD) and is usually affected (mutated) in most disease cases. Hence, treatment by delivering gp91phox as a transgene cures patients without major side effects. Prominent locations where ADA deficiency has been treated include the US, UK, and Italy, with commendable successes [9], though not 100% of the time. For example, in the case of some SCID-X1 patients treated by gene therapy, the enthusiasm was dampened as 2–6 years later an onset of acute T-cell lymphoblastic leukemia (T-ALL) was reported in 5 of 60 patients who received HSC as gene therapy in the past [10]. However, the situation was resolved or managed by the activation of LIN-11, Isl-1 and MEC-3 (LIM) domain only 2 (LMO2), a proto-oncogene, and a transcriptional cofactor capable of promoting the self-renewal of committed T cells [11]. The previous intended therapeutic use of gamma retroviral vectors (usually containing intact 50 long terminal repeats [LTRs]) produced severe adverse effects that were noticed in early implementation of gene therapy trials, calling for a search into the process of retroviral incorporation into human cell lines as well as primary human HSCs (CD34þ), which ultimately elucidated the need to search for better alternatives [12]. Even when using gene therapy methods, different outcomes have been recorded with different and distinct PIDs. This necessitates the establishment of protocols that consider the uniqueness of the different diseases, especially as they relate to vector design. This is actually dependent on the fundamental understanding of the molecular basis for interaction.

It is important to note that viral vector integration is actually an active process catalyzed by the tethering of the viral preintegration complex to the open chromatin regions in the genome of the host cell characterized by DNaseI hypersensitive sites as well as epigenetic marks [12]. Considering all the previous points so far, vector–chromatin interaction and its consequences have turned out to be appreciably more predictable; however, vector-induced leukemogenesis remains an unpredictable factor as it pertains to gene therapy, owing to its multifactorial nature. Nevertheless, there is a need to balance the glaring potential adverse effects against the clinical benefits for a particular patient, while putting the clinical complications associated with alternative treatments into perspective (e.g., allogeneic hematopoietic stem cell transplantation (HSCT) from a mismatched donor). The success along with the feasibility of gene therapy is undeniable, taking into account the benefits recorded with patients of different classes of diseases or disorders needing cure, including SCID-X1 and ADA-SCID, within the last two decades. PIDs (manifesting in the so-called bubble boys and girls) are rare without any doubt; nevertheless, they are life-threatening genetic diseases capable of severely compromising the integrity and functions of the immune system. This forces such individuals to live in a pathogen-free environment due to an inefficient immune system. PIDs targeted by gene therapy, which include ADA-SCID, SCID-X1, WAS, and CGD, occur in children with symptoms that include recurrent infections, system failure, and often death, most likely soon after birth. Bone marrow transplantation (using HSC) from human leukocyte antigen (HLA)-matched donors (<20% availability) has been a good alternative; however, the risks and complications associated with such strategies typically outweigh the benefits [13,14,15]. The alternative strategies that address these setbacks/limitations of scarce HLA-matched donors are found in the therapeutic potentials of autologous HSCs. As a result, this helps to avoid complications associated with immune suppression (IS) as well as the hurdle of graft against host infections [16,17,18,19].

Major setbacks and safety concerns are involved in the use of the existing murine γ-retroviral vectors designed for *ex vivo* gene transfer, leading to the onset of leukemia in a good number of patients who received treatment [20,21,22]. However, SCID-X1, which actually involves immunodeficiency disorders with T cells absent, natural killer (NK) cell deficiency, impaired function of B cells, and γ-chain (γc)-dependent cytokines, can be corrected by the reconstitution of immune competence in patients (for instance, via the application of SIN viral vectors). One of the core advantages of SIN is that they are devoid of LTR promoter/enhancer function, thereby reducing the risk of clonal dominance as well as insertion-related mutagenesis [23,24,25]. A similar application of the γ-retroviral vectors (murine) is present in ADA-SCID, a fatal PID with impaired T-, B-, and NK-cell growth, characterized by mutations in the gene coding for ADA (responsible for detoxifying the body of toxic purine metabolites), thus exposing patients to severe infections [9,16,17,18,26].

A number of ongoing clinical trials (#NCT01175239 (London), #NCT01410019 (Paris), #NCT01129544 (United States), #NCT01852071, #NCT01380990) are aimed at infusing interleukin-2 receptor γ-chain gene (IL2RG) to autologous HSCs in order to be transduced into SCID-X1 recipients to restore T-cell efficiency [26,27]. The same approach applies to WAS (#NCT01515462). There are also other areas where SIN-LVs are beneficially used. Autologous CD34+ HSCs derived from bone marrow, transduced *ex vivo* by means of LV-w1.6W vectors that convey a corrected copy of *WASp* gene, is one of the specific applications of LVs to WAS therapy. Compared with the γ-retroviral strategy for gene therapy in WAS, no clonal dominance or insertional mutagenesis was observed, and there is no need for a germ-free environment [28,29,30]. In addition to the gene correction in multiple cell lineages as well as the noticeably faster immunological reconstitution, the absence of detectable vector integration-associated adverse reactions is observed up to 3 years after close monitoring [31,32,33]. Approaches, such as employing a myeloid-specific promoter to avoid insertion-related mutation complications, are aimed at achieving better gene-corrected cell reconstitution and are also currently a critical area of focus. In these trials, however, some conditions could not be monitored effectively due to some other influencing conditions such as the metabolic effects of enzyme replacement therapy [9,16,34,35]. Low engraftment of gene-corrected cells in attempts to manage CGD (an uncommon genetic disorder because of a mutation in gp91phox subunit of nicotinamide adenine dinucleotide phosphate oxidase [NADPH]) has proven relatively unsuccessful due to the transient clinical benefits that it provides, as evidenced in the recurrence of the disease. The disease results in the failure of phagocytic cells (macrophages and neutrophils) to generate reactive oxygen species, hampering the efficient clearance of fungal and bacterial infections [36]. The use of γ-retroviral vectors for this disease also results in clonal dominance of gene-corrected cells capable of producing monosomy and myelodysplastic syndrome [27,36,37]. Most importantly, now is the time to put a spotlight on the most recent developments in this field as well as putting forth a road map for the future breakthroughs.

Vaccines are most commonly made up from the attenuated pathogens in forms administrable into a host’s system. However, some vaccines fail, and two main reasons could account for why vaccines fail after their preliminary development. The first is failure of the immune response, and the second is pathogenicity. The potency of a vaccine heavily relies on its ability to elicit an immune response just by recognition, while remaining nonpathogenic. However, the main cause of why vaccines fail, is that the vaccine candidate may not induce an immune response, as we lack full and comprehensive knowledge of the complexities of the host immune system. Second cause is the ability of the vaccine candidate to illicit an immune response by actually becoming pathogenic. In most PID cases, administration of subcutaneous or intravenous immunoglobulin is the primary choice of treatment. The main challenges with the use of vaccines in PIDs are the impairment of immune response, and impediment the immune system’s antigen stimulation, which results in very little or no protection caused by the immunodeficiency in some PID cases. In addition, adverse effects often result from vaccines as well as possible emergence of pathogenesis from vaccine strains. For example, in patients with phagocytic cell defects or defective T cells or NK cells (leukocyte adhesion deficiency), the immune response could be altered as a result of the live viral as well as live attenuated bacterial vaccines. This can create severe disorders linked to the vaccine strains, with the Chediak–Higashi syndrome and CGD being a common example.

### 2.1 Current developments en route for managing hemoglobin disorders such as sickle cell disease and β-thalassemia

β-Thalassemia and sickle cell anemia are two prominent monogenic blood hemoglobin disorders responsible for early mortality and morbidity. Mutation in the β-globin of hemoglobin is what gives rise to hemoglobin S, otherwise known as sickle hemoglobin, resulting in a “sickle-shaped” erythrocytes that lack flexibility, thereby obstructing blood flow by sticking to blood vessels and depriving cells of oxygen. Progress reports show that, where hydroxyurea with the purpose of increasing the expression of the fetal globin gene failed, *HSC* gene transfer of a β-globin mediated by LV has proven successful in the treatment of a child (13-year-old), producing a 47% β-globin expression when monitored for more than a year. The strategy is that the β-globin carrying a missense mutation that confers “anti-sickling” properties (βA-T87Q) on it [38,39]. β-Thalassemia stems from the loss of β-globin, a functional component of hemoglobin in red blood cells. The previously available treatment options (with the exception of bone marrow transplantation), such as frequent blood transfusions and chelation therapy to avert the amassing of iron, are not curative and have associated side effects. Complications of a matched donor arise with the bone marrow transplant option, in addition to the high risk.

Although challenges are involved in acquiring an adequate amount of corrected globin protein expressing genes in erythrocytes, curative gene transfer to HSC seems to be a good alternative. For instance, transduced autologous CD34+ HSCs via an SIN-LV coding for a functional copy of the β-globin gene has become a feasible procedure. However, myeloablative conditioning was necessary before reinfusing the gene-corrected HSCs. In addition to the phase 1/2 studies (#NCT01639690, #NCT02151526, #NCT02453477, and #NCT01745120), where LV transfer of an engineered β-globin gene to HSC has been used to treat patients (e.g., those with βE form) without the need for multiple transfusions and iron chelation therapy, recipients of such treatments are progressing healthwise for up to 2 years and beyond [38,40]. This treatment option is not as straightforward as in the case of the *β*_0_/*β*_0_ genotype, due to the lack of endogenous expression, resulting in only a partial correction of the disorder. Relentless efforts toward making a cure in the near future for many genetic diseases, including globin disorder, are also ongoing in different locations, including Thailand, the US, Italy, Australia, France, and other countries (Table 2).

**Table 2:** Various countries involved in the geographical distribution of gene therapy clinical trials in order of descending percentage hierarchy

Country	Gene therapy clinical trials	
	Number	Percentage
USA	1,550	62.9
UK	219	8.9
Multicountry	120	4.9
Germany	92	3.7
China	68	2.8
France	57	2.3
Switzerland	50	2
Japan	42	1.7
The Netherlands	36	1.5
Australia	32	1.3
Spain	29	1.2
Canada	27	1.1
Italy	26	1.1
Belgium	22	0.9
South Korea	20	0.8
Sweden	12	0.5
Russia	10	0.4
Israel	8	0.3
Poland	6	0.2
Finland	6	0.2
Norway	4	0.2
Austria	3	0.1
Singapore	3	0.1
Czech Republic	2	0.1
Denmark	2	0.1
Ireland	2	0.1
Mexico	2	0.1
New Zealand	2	0.1
Taiwan	2	0.1
Kenya	1	0
Kuwait	1	0
Gambia	1	0
Romania	1	0
Uganda	1	0
Burkina Faso	1	0
Egypt	1	0
Saudi Arabia	1	0
Senegal	1	0
Total	2,463	

Data sourced from: www.wiley.co.uk/genmed/clinical on June, 2, 2017.

### 2.2 Improvements for ocular disease

The AAV vectors find application in ocular gene transfer and have been extensively used in retinal diseases gene therapy (NCT02161380 and #NCT01267422), including inborn forms of blindness (e.g., Leber’s congenital amaurosis type 2 [LCA2]) for which no prior treatment existed [41,42]. Using a murine model in gene therapy efforts on Leber hereditary optic neuropathy has shown that a single intravitreal inoculation of scAAV2, having triple Y-F mutations in AAV2 capsid and conveying a wild-type human *ND4* gene, is able to further halt the degeneration of the optic nerve, accompanied by a significantly elevated proportion of complex-I-dependent ATP synthesis. This suggests the correction of the compromised electron transport chain (ETC). Mutations in the *RPE65* gene (expressing a 65-kilodalton protein) lead to retinal pigment epithelium inexpression, thereby impairing visual phototransduction. It is a mitochondrial gene affecting the complex I of ETC. Clinical studies (#NCT00643747, #NCT01208389, and #NCT00481546) have demonstrated that a single-dose subretinal injection of AAV2 vector to deliver the therapeutic gene (*RPE65*) can improve vision [41,43,44,45,46,47,48]. Through well-modulated final formulation, vector design, and immunomodulatory regimens, long-term follow-up on these clinical studies suggests an improvement in the visual acuity, sensitivity of the retina, and gain of function over time [49]. Canine and murine models have proven that gene amplification could limit the progression of degeneration if the intervention starts early [50,51,52,53]. Accomplishment with the LCA2 gene therapy is a milestone for clinical trials in other retinal diseases (#NCT01461213; for choroideremia) [54]. This genetic disease results from a nonfunctional copy of the *CHM* gene. Obvious diseased manifestations are slow and advance deterioration of the patient’s choroid, photoreceptors, and retinal-pigmented epithelium. This could result in the complete loss of sight at an intermediate age. However, a subretinal administration of the AAV2 vector conveying the *CHM* gene significantly improves the vision.

### 2.3 Revisiting neurodegenerative disorders

The central nervous system is the most complex of all the systems in the body, and as such, treating neurodegenerative disorders with conventional pharmacological medications is very difficult. The complexity is made obvious in the blood–brain barrier (BBB), which could restrict even the most therapeutically potent agent from getting access to the target site. Although molecular gene therapy seems to solve this difficulty, the challenges of vector delivery to the CNS-specific target site persist. This notwithstanding, successes have now been reported for cases involving metachromatic leukodystrophy (in which patients are deficient in arylsulfatase A [ARSA], leading to the accumulation of sulfatide in myelin-producing cells that produce severe cognitive and motor damage), aromatic l-amino acid decarboxylase (AADC) deficiency, and adrenoleukodystrophy (ALD, with mutated ABCD1 gene, affecting the adrenal cortex). These procedures employ LV (integrating) and nonintegrating AAV without any reported major safety concern [55]. Unexpectedly, neither HSC nor bone marrow transplant can effectively solve this challenge. It is, however, currently hypothesized that overexpression of ARSA in genetically modified hematopoietic cells could possibly remove these barriers associated with bone marrow transplant through the delivery of ARSA to stop the progress of demyelination (#NCT01560182), employing SIN-LV-mediated gene transfer in autologous CD34+ HSCs. A continuous production of the functional ARSA protein with normal cognitive and motor development without established insertion-associated mutagenesis has been reported. AADC deficiency results in impairment of the synthesis as well as secretion of neurotransmitters, including dopamine and serotonin. This leads to setbacks characterized by dystonia, oculogyric crises, truncal hypotonia, severe movement disorders, sweating, tongue protrusion, neurodegenerative impairment in children, and jaw spasms.

A gene therapy clinical trial (#NCT01395641) on AADC through direct injection of AAV2 vector to deliver *AADC* gene within the bilateral putamen, with obvious restoration in the patients, is currently in progress. Commendable scores on the Peabody Developmental Motor Scale, Alberta Infant Motor Scale, toddlers scores, and comprehensive developmental inventory support this for infants after 15–24 months of gene therapy [56,57]. Transient increases in dyskinesia and frequent episodes of apnea were noticeable adverse effects. Other clinical trials (e.g., #NCT00229736 and #NCT02122952), along this line in relation to Parkinson’s disease, spinal muscular atrophy type 1, and Canavan disease, are ongoing [58]. Other limitations associated with gene therapy applications to neurodegenerative disorders, such as unintended binding to extracellular matrix components and profuse spread from the injection site, have been observed. Capsid engineering, cisterna magna, and serotype alternatives are viable means of alleviating these common challenges [59,60].

### 2.4 Better approach to hemophilia B coagulation disorder

A very common well-known blood disease is hemophilia, a hematological disorder caused by mutations in the gene that codes for coagulation factor VIII or IX. The disorder is X-linked and occurs in about 1 in 5,000 (hemophilia A) or 1 in 30,000 (hemophilia B) male births throughout the world. In very simple and straightforward terms, the blood refuses to clot. Many possible consequences of these mutation errors could be deduced clinically. Contemporary treatment available for hemophilia involves very tedious and regular (about 2–3 times per week) intravenous infusion of recombinant or FVIII/FIX proteins (both of which are secreted in inactive form at plasma levels of 200 and 5,000 ng/mL, respectively) derived from the plasma. Such lifelong disease management is burdensome as well as expensive, and the third-world countries often cannot afford them. Gene therapy, on the other hand, is curative and sustained (greater than 10 years in canine models) following a single round of gene transfer. The gene therapy option for hemophilia remains a viable alternative because of the amount of cell types that are capable of synthesizing biologically active FVIII (synthesized basically in endothelial cells, including liver sinusoidal) and FIX (fundamentally synthesized in the hepatocytes) following gene transfer.

By using AAV vectors *in vivo*, molecular gene transfer through the hepatic artery of the liver can correct hemophilia B (FIX deficiency) and is considered an ideal strategy as demonstrated in preclinical studies [61,62]. However, additional studies showed that memory CD8+ T cells and neutralizing antibodies guard against the AAV capsid (particularly serotype 2) in humans who have had previous natural exposure to AAV, thereby eliminating the therapeutic transduced hepatocytes and obstructing gene transfer to the liver beyond a particular titer [63]. These points also highlight the fact that the immune system remains a challenge for *in vivo* gene transfer, noting that, in contrast to adenovirus, unaided AAV vectors do not incite strong immune responses. Clinical trials (e.g., #NCT00979238) have been initiated and executed on AAV8 (a different AAV serotype) with an abated production frequency of AAV neutralizing antibodies [64,65]. This strategy also provides for a less invasive peripheral vein administration by taking advantage of the self-complementary genome, a codon-optimized F9 sequence. This option provides the advantage of a transient IS regimen using prednisolone if the patients are showing mild transaminitis or loss of circulating FIX [66,67]. Hyperactive FIX variant (R338L, also FIX-Padua) occurring naturally in a self-complementary AAV8 vector is also in the pipeline of phase 1/2 molecular gene therapy testing (#NCT01687608) for treating hemophilia using animal models [68,69,70,71,72,73]. A long-term expression with enhanced catalytic action of FIX variant (FIXR338L) at reduced curative intended vector doses (scAAV8-FIXR338L) was observed [74,75]. Compared to the IS using prednisolone, which could not salvage expression, the subjects who were administered the highest vector dose lost expression, expressing IFN-ϒ producing T cells and transaminitis in response to the viral capsid antigen, though no liver toxicity was observed. Over time, no signs of patients’ emerging immune response were observed as a countermechanism against the FIX protein in more than 20 hemophilia B patients who have received AAV-F9 vector treatments, with additional research backing up these claims. However, some inconsistencies are observed with different clinical trials (Table 1), potentially related to issues such as vector design, vector doses, use of suppressive steroid drugs, higher transduction efficiency, the occurrence of immune stimulatory CpG motifs, or other factors for which answers and explanations are necessary [76,77,78,79,80,81,82,83].

## 3 CRISPR-Cas9 and the future of gene therapy

The CRISPR-Cas9 genome editing tool has recently become a common preference for gene therapy studies in basic research as well as in clinical trials. Though discussions are still ongoing concerning ethics in human applications, the CRISPR-Cas9 technology is undoubtedly a powerful tool for gene therapy when fully refined. CRISPR-Cas9 is an efficient site-directed genome editing tool mainly composed of Cas9 RNA-guided nuclease and CRISPR RNA (crRNA), which directs the Cas9 enzyme to the sequence of interest in the genome. This specificity is determined by a 20-nucleotide sequence complementary to the crRNA, following a protospacer adjacent motif sequence [84,85]. The CRISPR system uses the host cell’s innate error-prone nonhomologous end joining to achieve the aim. Many concerns regarding off-target and associated complications are still under review by experts to ascertain the safety of this genetic tool before it can be implemented in human trials. Genetic engineering and biotechnology companies are keeping a close look at the CRISPR-Cas9 tool as the technology has been used very recently in basic science research, and clinical trials are commencing in pharmaceutical research to use the CRISPR-Cas9 to treat patients with β-thalassemia [86].

Although CRISPR-Cas9 has proven to be an enormous scientific breakthrough, it has many limitations that have made it a frontline topic of discussion in research and medical applications, with legal implications. In 2015, an international summit on human gene editing supported the establishment of an international committee to assess the implications of CRISPR-Cas9. The Committee on Ethics, Law and Society of the Human Genome Organization on CRISPR-Cas9 produced some vital ethical points to consider in the application of CRISPR-Cas9, most particularly in its application to human biology and medicine [87]. On the bright side, CRISPR targets almost any nucleotide sequence with a short length of specific genes; however, it does not faithfully insert new target DNA sequences. Off-targets often occur, creating deleterious effects that are sometimes irreversible. This error leads to serious mutations such as in Friedreich ataxia, fragile X syndrome, and Huntington disease. As effective as it is, the CRISPR system can fail in identifying the right target, including introns, and as a result cause further damage. Challenges and ethical considerations about the use of CRISPR technology are not just peculiar to CRISPR but common to all gene editing tools previously employed. Experiences and challenges with gene editing raise discussion on delaying the application of the CRISPR-Cas9 in medical applications until well proven and tested using highly supported basic science [88,89,90,91]. For instance, an example is the emergence of childhood leukemia arising when the viral vector activated a latent human oncogene, as Double Strand Breaks (DSBs) generated by CRISPR-Cas9 often induces apoptosis. Some of the components of the CRISPR-Cas9 system, such as the cas9 protein, are of microbial origin and may trigger immune responses in the human system, which may be undetected for a longtime. The CRISPR-Cas9 system has been around only for a short time and requires more time to investigate all the implications associated with its use in genetic engineering.

## 4 Conclusions

In recent times, the field of gene therapy has recorded clinical achievements in various disease types by employing different methodologies and improved vectors. Recently, numerous gene therapy trials have validated the clinical benefits of the application of molecular biology and biotechnology tools in treating diseases or disorders that have proven elusive to cure using conventional pharmacotherapy. Such areas of application include hereditary immune system disorders, congenital eye diseases, hemophilia B, lipoprotein lipase deficiency, and adoptive transfer of genetically engineered T cells for cancer. Although gene therapy possesses obvious scientific, ethical, and technological challenges, many efforts have been made to facilitate the effectual translation of gene therapy into clinical practice. Both recent and noteworthy is the application of gene therapy to PIDs, hemoglobin, hemophilia B, ocular, and neurodegenerative disorders. Most recently, but still under strict refining, is the CRISPR-cas9 gene-editing tool; although it currently has some setbacks, it is promising at providing solutions to many genetic diseases. The application of gene therapy to these areas has pushed medical research and other applications toward becoming the remedy we have sought concerning such disease areas.
